# Phthalocyanines and Porphyrins/Polyaniline Composites (PANI/CuPctBu and PANI/TPPH_2_) as Sensing Materials for Ammonia Detection

**DOI:** 10.3390/polym14050891

**Published:** 2022-02-24

**Authors:** Alain Pauly, Sahal Saad Ali, Christelle Varenne, Jérôme Brunet, Eduard Llobet, Amadou L. Ndiaye

**Affiliations:** 1 Clermont Auvergne INP, CNRS, Institut Pascal, Université Clermont Auvergne, F-63000 Clermont-Ferrand, France; alain.pauly@uca.fr (A.P.); sahal@gmail.com (S.S.A.); christelle.varenne@uca.fr (C.V.); jerome.brunet@uca.fr (J.B.); 2 Department of Electronic Engineering, Microsystems Nanotechnologies for Chemical Analysis (MINOS), Universitat Rovira i Virgili, 43007 Tarragona, Spain; eduard.llobet@urv.cat; 3 CNRS, Institut Pascal, Université Clermont Auvergne, F-63000 Clermont-Ferrand, France

**Keywords:** polyaniline, phthalocyanine, porphyrins, ammonia, resistive sensors, humidity, temperature

## Abstract

We combined a conducting polymer, polyaniline (PANI), with an organic semiconducting macrocyclic (MCs) material. The macrocycles are the phthalocyanines and porphyrins used to tune the electrical properties of the PANI, which benefits from their ability to enhance sensor response. For this, we proceeded by a simple ultrasonically assisted reaction involving the two components, i.e., the PANI matrix and the MCs, to achieve the synthesis of the composite nanostructure PANI/MCs. The composite nanostructure has been characterized and deposited on interdigitated electrodes (IDEs) to construct resistive sensor devices. The isolated nanostructured composites present good electrical properties dominated by PANI electronic conductivity, and the characterization reveals that both components are present in the nanostructure. The experimental results obtained under gas exposures show that the composite nanostructures can be used as a sensing material with enhanced sensing properties. The sensing performance under different conditions, such as ambient humidity, and the sensor’s operating temperature are also investigated. Sensing behavior in deficient humidity levels and their response at different temperatures revealed unusual behaviors that help to understand the sensing mechanism. Gas sensors based on PANI/MCs demonstrate significant stability over time, but this stability is highly reduced after experiments in lower humidity conditions and at high temperatures.

## 1. Introduction

The development of gas sensors continues to gain interest since monitoring and detecting pollution and industrial emissions has become essential for environmental and public health agencies. Increasing efforts and new developments have been successfully promoted by recent progress in nanotechnology and chemical engineering. The modulation of material properties can be achieved either by surface engineering for tuning reactivity towards target species or by surface nano-structuration, which results in increased surface area, and thus higher sensitivity.

In another trend related to the emergence of renewable energy technologies, the development of sensors is more and more implemented since the production of renewable energy can be accompanied by the release of toxic pollutant gases such as ammonia (NH_3_) [1], hydrogen sulfide (H_2_S) [2], etc. Ammonia gas detection in this field is not only essential in terms of security, e.g., occupational exposure to ammonia in bio-energy plants needs to be monitored, but also in terms of production efficiency. Ammonia is a limiting factor for the bio-energy yield (efficiency). So, the development of ammonia sensors especially dedicated to these areas must also be considered. In bio-energy production, high humidity levels and temperatures are inherent factors that must be taken into consideration when developing sensors. Such harsh operating conditions make sensor material research in this field more challenging. If sensitivity is often the main metrological performance to reach, sensor response stability under such conditions is even more important.

For the development of ammonia gas sensors, a wide range of materials, such as solid-state metal oxides, conducting polymers, and nanocarbonaceous materials, has already been investigated. Recently, hybrid materials made of these categories have emerged as new and challenging structures for the development of innovative sensors. The frequently used conjugated polymers for developing gas sensors are polyaniline (PANI), polypyrrole, and polythiophene [3,4,5]. PANI has been investigated as a potential sensing material due to its controllable electrical conductivity, reversible redox behavior, good sensitivity at room temperature, good environmental stability, and relative ease of processing. PANI can be combined with different sensing matrices, such as carbon materials [6,7,8]. In addition to these combinations, PANI can serve as a sensing matrix to host metal oxides [9,10,11,12,13], or functional units [14], allowing for the exploration of other application areas. The advantages of using conducting polymers as sensitive materials compared to inorganic materials are their natural conductivity, fast response, low cost, easy synthesis, and sensitivity at room temperature. Polyaniline presents different oxidations states, leading to structures that are chemically and physically different from each other. Such properties make PANI a material suitable for gas sensing applications [4,14,15].

The combination of PANI with other additives (metal oxides, nanoparticles (NPs), carbon nanotubes, or graphene etc.) allows for the tuning of sensing properties and, particularly, for enhancing specificity. While examples of the combination of PANI with metal oxides, NPs, carbon nanotubes, graphene, etc. are found in the literature, its combination with organic semiconductors such as phthalocyanines and porphyrins is less exploited [5,16,17,18,19,20,21,22,23]. Examples of these PANI-macrocycle combinations dedicated to gas sensors are also rare [5,19,23]. Knowing that these MCs are potentially well documented [24] for their capacity to enhance sensitivity, we have focused on such a combination with PANI. Macrocycles are highly resistive materials (approaching quasi-isolating material). Their combination with PANI can help to produce highly resistive materials, which enables higher resistance variation under gas exposure. Such higher resistance variation is beneficial for improving sensitivity. Additionally, their intercalation into the PANI matrix will certainly limit the harmful effects that high ambient humidity or temperature can have on the polymer. Therefore, besides achieving an improved sensitivity, we intend to evaluate such nanostructures for their ability to withstand higher temperatures and different humidity environments for sensing ammonia.

This article presents the combination of a conducting polymer (PANI) with an organic semiconducting material (MCs) using a simple ultrasonically assisted reaction. The MCs possess peripheral groups (tetra tert-butyl and phenyl), ensuring good solubility in reaction media. The nanostructured composites developed have been deposited on interdigitated electrodes (IDEs) and used as resistive sensors. We will focus on the electrical characterization of the composite nanostructure PANI/MCs performed using the I–V (current–voltage) characterization process. The sensing performance of this composite towards ammonia will be discussed by focusing primarily on the stability and the responses in a high temperature and high humidity environment.

## 2. Materials and Methods

### 2.1. Materials and Solvents

Acetonitrile, methanol, and chloroform were purchased from Aldrich (Lyon, France) and used as solvents. Aniline, sulphuric acid [H_2_SO_4_ (0.5M)], ammonium peroxodisulfate [(NH_4_)_2_S_2_O_8_], and copper (II) 2,9,16,23-tetra-tert-butyl-29H,31H-phthalocyanine (purity 97%) and 5,10,15,20-tetraphenyl-21H,23H-porphine (purity 97%), denoted as CuPctBu and TPPH_2_, respectively, were obtained from Aldrich and used without further purification.

### 2.2. Materials Preparation

#### 2.2.1. PANI Synthesis

For the preparation of the conducting polymer, we used a previously reported procedure [25] to obtain acid-doped conducting PANI. According to this synthesis process, the oxidative polymerization method based on ammonium persulfate and sulphuric acid (H_2_SO_4_) as a dopant is used to prepare polyaniline. In an example of synthesis, 25 mL of a diluted H_2_SO_4_ (0.25 M) solution was added to 10 mL of aniline under stirring and cooled using an ice bath (0 °C), which gave rise to a white, salt-like mixture. This mixture was dropwise added to an acidic solution of ammonium persulfate (31.26 g in 30 mL H_2_SO_4_). The white dispersion turned brown after the first drop and ended up with a green coloration at the end of the process. The resulting mixture was allowed to remain under stirring for 24 h. The mixture was then filtered and washed with acidic water (100 mL H_2_O + 50 mL H_2_SO_4_ 0.5 M) before being stored at room temperature. The resulting PANI powder could be re-dispersed in acetonitrile for the coating process.

#### 2.2.2. PANI/MCs Nanostructured Composite Preparation

To prepare the nanostructured composite (MCs/PANI), we adapted the preparation method developed for the non-covalent functionalization of carbon material already published [24]. In this process, conducted at room temperature, we dispersed PANI in acetonitrile (2 mg/mL, preparation based on 5 mL) by employing an ultrasonic bath for 5 min. We added a chloroform solution of the macrocycle (0.8, 1.6, 2.4 mg/mL), still under ultrasonic treatment, and performed an additional treatment for 10 min. Decantation overnight allowed the material to become sedimentary, and the resulting solids were washed thoroughly and redispersed in acetonitrile before deposition on IDEs.

### 2.3. Characterization Methods

Unless otherwise stated, all characterizations were conducted at room temperature. UV-visible spectra were recorded on a Lambda 2S Perkin-Elmer spectrometer. For the UV-vis spectra recording, a 1 cm path length glass cell was used, and the nanostructured composite concentrations were adjusted to 0.0005 mM with acetonitrile as a solvent. Raman spectra were recorded with a Jobin Yvon T64000 spectrometer with a charge-coupled device multichannel detector. Raman spectra were acquired at room temperature using an argon laser’s excitation wavelength of 514 nm.

Electron microscopy (SEM) micrographs were obtained from a JEOL 6060 low vacuum operating at 5 kV. The samples were prepared by drop-casting the composites on copper substrates, followed by drying at room temperature.

For the electrical characterization (I–V curves), the current (I) versus applied voltage (V) measurements were performed in ambient air employing a Keithley 2636 System Source Meter, which is controlled by LabVIEW software (version 2021). Generally, the electrical measurements were recorded under room temperature conditions in a two-point configuration. The I–V curves were obtained within a −1 to 1 V range (for a higher range, see Supporting Information) at a step voltage of 0.01 V. Temperature-dependence measurements of the sensor devices were carried out by adapting the measurement chamber with a cryostat device (filled with liquid nitrogen), enabling operating temperatures from 100 to 500 K.

#### 2.3.1. Sensor Devices and Sensor Preparation

The resistive sensor transducer consisted of interdigitated electrodes screen-printed onto an alumina substrate. The electrodes were made of platinum and presented 5 mm × 3 mm (length × width) geometry, with an interelectrode distance of 125 μm. The IDEs were also equipped on their backside with a meander made of platinum to ensure heating for operating the sensor at higher temperatures.

For the elaboration of the resistive sensors, IDE substrates were installed on a hot plate set at 80 °C to ensure rapid solvent evaporation. Then, 5–10 µL of the material was deposited on the IDEs, which were connected to an electrical measurement set-up that used a digital multimeter (Keithley model 2000) apparatus to follow resistance evolution during layer formation.

#### 2.3.2. Gas Sensing Experiments

The gas exposure experiments were performed using a dilution bench, which consisted of pollutant sources, the exposure chamber, and a computer-assisted data acquisition and monitoring program through LabVIEW. The gas source (gas cylinders) was diluted with dry air (relative humidity (RH) ~3–5%) to obtain the desired concentration range for a typical experiment. For the temperature experiment, the heating-controlled devices connected to the backside of the IDEs allowed for operating the sensors at the desired temperature. A humidity control unit was adapted to the test bench to provide a controlled RH that could range between 10% and 70%. We used a digital multimeter (Keithley model 2700) for monitoring the sensor resistance variation under gas exposure and recovery steps. If not otherwise indicated, exposure sequences were 15 min and recovery was 45 min, with sensors operated at room temperature, which was found to be in the 25–28 °C range. The experimental bench is displayed in Figure S1 (see Supporting Information).

For the resistive sensor analysis, sensor responses are represented by either ΔR or ΔR/R_0_ values, using the following formula:

ΔR/R_0_ = (R_Gas_ − R_0_)/R_0_ where ΔR = R_Gas_ − R_0_ (1)

with resistance under purified air (R_0_) and resistance under gas (R_Gas_).

It is worth noting that this ΔR/R_0_ value can also be expressed as a percentage by multiplying it by a factor of 100.

The sensitivity (S) of the sensors is defined as the sensor response per unit concentration (ppm), as given in the following formula: S = ΔR/R_0_ (%)/Concentration (ppm).

The sensitivity can be, therefore, expressed in %/ppm. Response time (τ_resp_) and recovery time (τ_rec_) are the times needed to reach 90% of the maximum response magnitude and recover 90% of the background signal, respectively.

## 3. Results

### 3.1. Materials Characterization

#### 3.1.1. Effect of the Functionalization on the Electrical Characteristics

**Figure 1:**
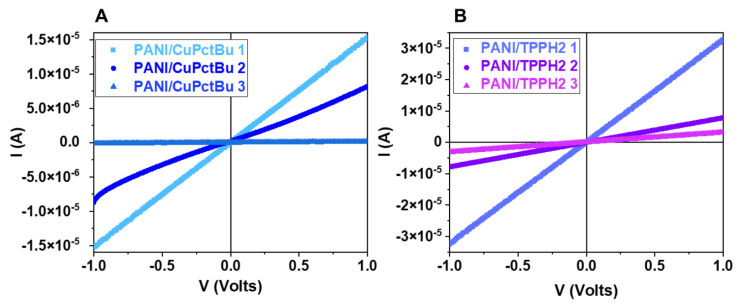
Current-voltage characteristics of the PANI/CuPctBu (**A**) and PANI/TPPH_2_ (**B**) composites at different MCs concentration.

In the first experiment, we tried to find a relationship between the degree of functionalization and electrical characteristic. We performed an experiment in which the amount of PANI was fixed while varying the concentration of macrocycles. For a typical case, 0.8 mg, 1.6 mg, and 2.4 mg of macrocycles in chloroform (1 mL) were added to a dispersion of PANI (2 mg/mL based on 5 mL solution), and an ultrasonic procedure similar to that developed for the preparation of the composite (explained above) was applied. The I–V curves for the three resulting devices (denoted PANI/MCs 1, PANI/MCs 2, and PANI/MCs 3, corresponding to the addition of 0.8 mg, 1.6 mg, and 2.4 mg, respectively) are presented below in Figure 1. The exact amount of dispersion (10 µL) was used in each case to ensure repeatable results. This volume is relatively high compared to what we usually employ to prepare resistive sensors. Indeed, thicker sensing layers lead to mechanical instability and lower resistance. But in this case, it was necessary to use such thick layers for the characterization.

The electrical characteristics (for PANI/CuPctBu and PANI/TPPH_2_) show a linear and ohmic behavior (Figure 1). The results are correlated with the degree of functionalization. The more the functional moieties (concentration), the higher the slope of the I–V curve, i.e., the less resistive are the devices. This result is not surprising since the MCs are highly resistive materials (quasi-insulating) and can lower the global resistance when wrapped or mixed with conducting PANI. The I–V characterization was also performed in the higher voltage range (Figure S2). Still, the tendency was identical, showing that the resistance is tunable by adding and wrapping the PCs around PANI. We can use these methods to build highly resistive PANI-based nanostructures.

In sensing films, the kinetic parameters are often diffusion dependent. Therefore, it is essential to develop thin layers to get more reliable responses, since thicker layers often experience diffusion phenomena or instability. In the case of these insulating macrocycles wrapped on the PANI, the situation is different since they can, themselves, serve as a binding agent and ensure a compact structure. Therefore, more material is needed to achieve good resistance variation for the preparation of the resistive sensors.

#### 3.1.2. UV-Vis Spectroscopy Characterization

**Figure 2:**
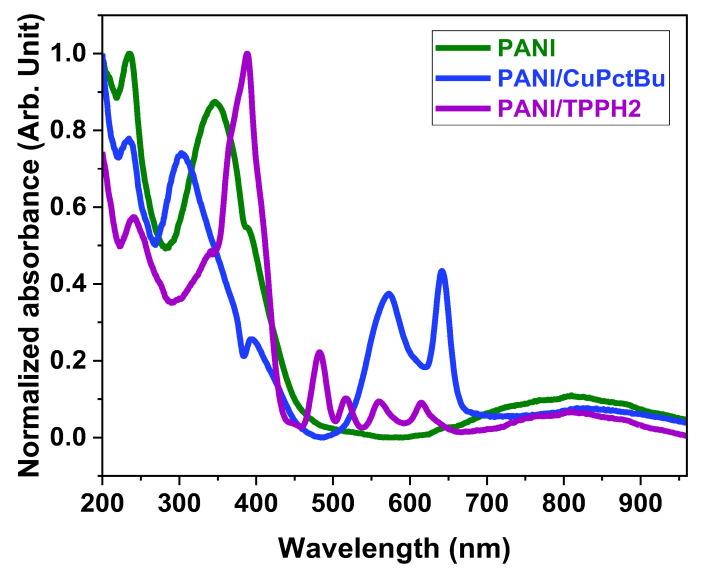
Normalized UV-Vis spectra of the different PANI composites. For clarity, the spectrum of PANI is also presented.

The UV-Vis spectra of PANI composites (PANI/MCs) are compared to that of PANI and presented in Figure 2. For PANI/CuPctBu, the spectrum shows a typical Soret band around 337 nm, corresponding to the π → π* transition of the metal phthalocyanines, and two bands located at 602 nm and 673 nm corresponding to the so-called Q bands illustrating the metal phthalocyanine signature [24]. The shoulder around 428 nm is typically observed in PANI-based derivatives and is attributed to transitions involving polaron states. The additional broad peak at 700–900 nm, also attributed to PANI, is due to the high doping level [19,26]. Finally, the peak at 330 nm in PANI/CuPctBu is attributed to PANI.

For the PANI /TPPH_2_ composite, the same conclusion can be drawn: peaks at 419 nm (B bands) and 515, 550, 592, and 641 nm (Q bands) are attributed to the TPPH_2_ [24] and the other to PANI. The shoulder located at 421 nm in PANI does not appear in PANI/TTPH_2_. This is presumably due to it having been enveloped into the intense Soret (at 419 nm) band of the TPPH_2_.

The UV-Vis spectra clearly established the presence of both components (PANI and MCs), thus confirming the formation of the nanostructure composites. In many given literature examples, the peak at 420 nm provides an indication that the PANI is in salt form (doped) since this peak shows the formation of the conducting state [26]. These observations are in accordance with the conductive form of the prepared samples.

#### 3.1.3. Raman Characterization

**Figure 3:**
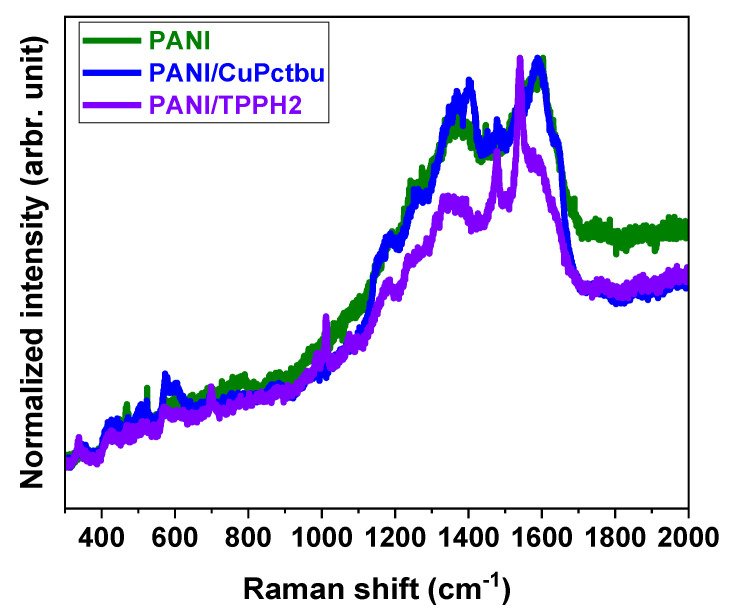
Normalized Raman spectra of the different PANI composites. For clarity, the spectrum of PANI is also presented.

For a better comparison, the Raman spectrum of the PANI matrix has been added to the study, as presented in Figure 3. In the Raman spectrum of PANI, mainly two major peaks are identified: the Raman broad peak positioned at 1590 cm^−1^ is assigned C–C vibrations of the pyrrole chemical group, whereas the broad peaks located at 1360 cm^−1^ can be assigned to isoindole moieties. The first signal (1590 cm^−1^) is attributed to the stretching vibration from the quinoïd ring. It is essentially due to the protonation of the polymer backbone and the semiquinonoid ring formation [27]. However, the signal at 1336 cm^−1^ is attributed to the vibration mode of the delocalized polaron structure. In PANI/CuPctBu and PANI/TPPH_2_, the previous peaks are overlapped with peaks essentially coming from the CuPctBu and TPPH_2_. The peaks at 1367 cm^−1^ and 1401 cm^−1^ in PANI/CuPctBu indicate C–N isoindole ring stretching [28] and are superimposed with stretching vibration from the PANI quinoïd ring. The peak at 579 cm^−1^ indicates the out-of-plane bending vibration of the CuPctBu [29].

In the case of PANI/TPPH_2_, the spectrum also shows an additional peak attributed to the TPPH_2_. These peaks are situated at 1540 cm^−1^ and 1478 cm^−1^, corresponding to the C–C vibration of pyrrole groups [30,31], while the peak positioned at 1011 cm^−1^ is attributed to the pyrrole C–N breathing mode [30]. All this information indicates the formation of the composite structure made of PANI and MCs.

#### 3.1.4. Scanning Electron Microscopy (SEM) Characterization

**Figure 4:**
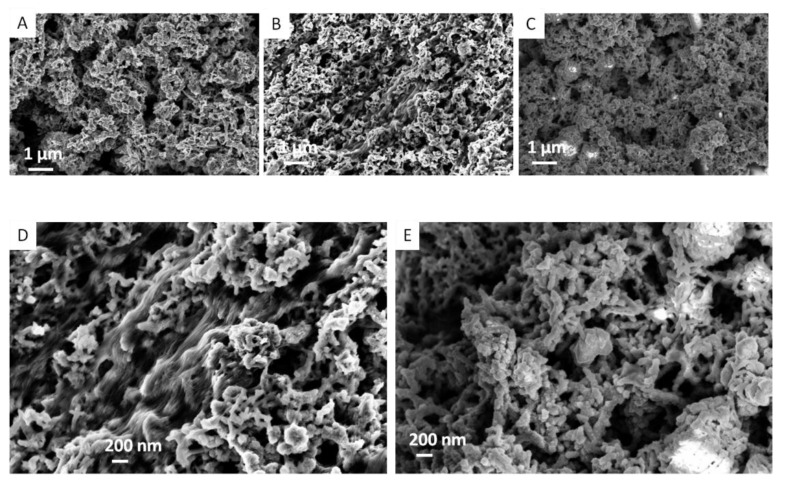
SEM images of PANI (**A**), PANI/CuPctBu (**B**), and PANI/TPPH2 (**C**), and higher magnification images for PANI/CuPctBu (**D**) and PANI/TPPH2 (**E**).

SEM characterization for PANI/CuPctBu and PANI/TPPH_2_ showed fiber-like nanostructures, as displayed in Figure 4. The PANI revealed fibrous structures, whereas the macrocycles are differently organized, depending on their type. The images also show that such a fibrous nature potentially allowed the formation of a porous structure, as seen in the higher magnification images.

As highlighted at higher magnification (Figure 4D,E), the CuPctBu are wrapped around the PANI fibers and form a wire-like network linking the PANI fibers, while the TPPH_2_ is revealed to be aggregate-like (Figure S3), forming a less compacted structure. The analysis of these images shows a compact design where the macrocycles work as a binding agent within the films. Figure 4D,E reveals the interconnected, fiber-like chains with the presence of both intra- and inter-chains. These interlaced structures suggest that conductivity will be governed by both intra- and inter-chain conductivity.

#### 3.1.5. Electrical Characterization

**Figure 5:**
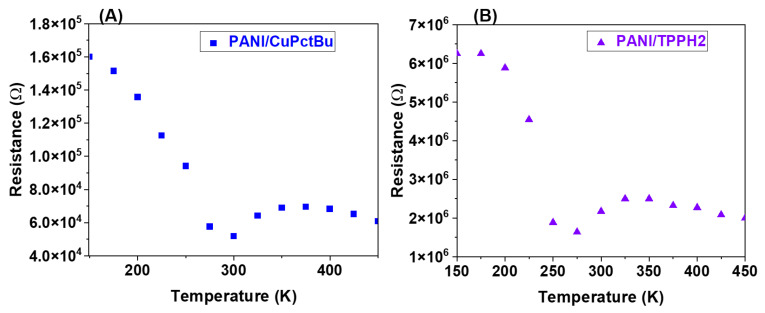
Temperature-dependent resistance variation of the PANI/CuPctBu (**A**) and PANI/TPPH_2_ (**B**) composites. Resistance is calculated from current–voltage measurement at the given temperature.

We performed temperature-dependent current–voltage measurements to analyze the conduction mechanism in PANI/MCs composites by recording the resistance variation (Figure 5). As in the case of PANI [25], the characterization of the nanostructured composites presents both positive and negative temperature coefficient resistance (TCR) [32,33]. The temperature-dependent current–voltage measurements for PANI are presented in Figure S4 for comparison. In the case of PANI/CuPctBu, the negative TCR occurs within the 150 to 300 K range, while in PANI/TPPH_2_ this occurs between 150 and 275 K. In the lower temperature range, the composite films show negative dR/dT, indicative of nonmetallic behavior, while in the higher temperature range, a positive dR/dT indicative of metallic behavior is observed. Even a slight decrease (negative dR/dT) is observed in the 350–450 K range. This behavior has already been observed and explained as a mix of different conduction states [33,34].

In the present case of PANI/MCs, we can also expect a mix of metallic and nonmetallic conduction mechanisms. This mixed conduction mechanism would be responsible for such resistance evolution inversion (positive and negative evolution). This confirmation of mixed metallic-nonmetallic character in terms of conductivity is expected since, in the nanostructured composites, PANI governs conductivity. This observation shows that even by inducing a higher resistance in the composites, the macrocycles do not alter the conduction mechanism in the composite.

### 3.2. Gas Sensing Performance

#### 3.2.1. Typical Sensor Response toward Ammonia Exposure

**Figure 6:**
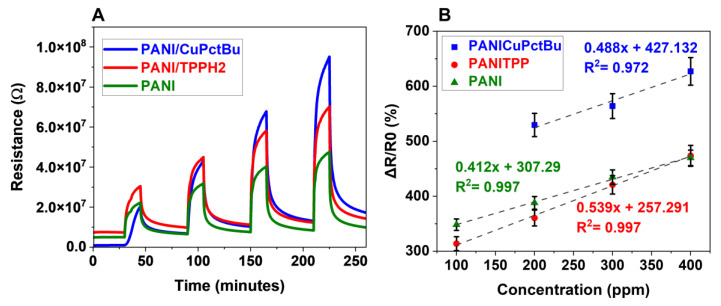
The resistive sensor response of PANI, PANI/CuPctBu, and PANI/TPPH2 (**A**), and the calibration curves corresponding to ammonia exposure in the 100–400 ppm range (**B**) at room temperature.

Figure 6A presents the typical response–recovery characteristics measured at room temperature for PANI-based nanostructured composites. The study has been conducted with sensors prepared from the PANI/MCs 2 (see the experimental section) dispersion preparation. These sensors have been exposed to different concentrations of ammonia ranging from 100 to 400 ppm. For clarity, the response of PANI is also added for comparison. Both sensors, PANI/CuPctBu and PANI/TPPH_2_, showed a relatively high response and reversible characteristics, concomitant with the ammonia exposure leading to resistance increase due to deprotonation. At the same time, recovery in air follows the reverse reaction (protonation in air). Apart from the first exposure cycle of PANI/CuPctBu, which presents an unusual trend (steady state is not achieved), all exposure/recovery cycles are reversible. However, the sensor layers show a slight baseline up-drift, which is more critical in PANI/CuPctBu than in PANI/TPPH_2_. The incomplete desorption during the cleaning periods can explain this result after ammonia exposure. Based on the calibration curves, we can see that the addition of the MCs into the PANI matrix improves the sensing behavior.

Figure 6B presents the calibration curve, calculated from Figure 6A and represented by ΔR/R_0_ using the formula given in the experimental part. A linear trend is observed with PANI/CuPctBu, presenting the slightly lower sensitivity, even with a higher response than seen in PANI/TPPH_2_. The sensitivities extracted from the calibration curves are 0.54%/ppm for PANI/TPPH_2_ and 0.49%/ppm for PANI/CuPctBu (almost identical), with the PANI/TPPH_2_ presenting the better trend as attested by its R^2^ value of 0.99 compared to 0.97 for PANI/CuPctBu. Overall, the sensitivity of the PANI/MCs is better than that of PANI (0.41%/ppm) itself, illustrating an improvement in the sensing performance. These results show that wrapping the PANI with macrocycles that are insulating in nature has no limiting effect on the conductivity of the matrix.

Response time and recovery time are calculated from the dynamic response curves based on the definitions given in the experimental part. The response times are measured to be 510 s for PANI/CuPctBu, and 480 s for PANI/TPPH_2_, while the recovery times are measured to be 990 s and 1080 s for PANI/CuPctBu and PANI/TPPH_2_, respectively. The responses times are slightly longer than in the case of PANI itself (420 s). This can be attributed to the presence of the MCs that do not favor rapid kinetics within the structure. The recovery time is, however, slightly longer in the case of PANI (1200 s) compared to the PANI/MCs. The faster desorption observed in composites relies on the lower reactivity of the PANI/MCs structures, which are more compact than PANI, as seen in the SEM images.

Table 1 summarizes the reported examples close to our case, since comparable and reliable models are hard to find. These examples concern literature data on PANI and related composites approaching our sensors.

From this table, it is clear that our sensors provide a better response (with ΔR/R_0_ > 300%) than the others reported in Table 1. But, in terms of response and recovery times, our sensors present longer times than the reported ones. This observation can be stated as an opportunity to improve synthesis and favor rapid kinetics. It is also worth noting that exposure times are relatively short in some of these examples, meaning that the steady state is sometimes not achieved [39]. The short response times reported by some studies in Table 1 are explained by either the ultra-thin film structure [35], the combination of the different elements of the composites [37], or the synergetic effect of the protonation process [40].

#### 3.2.2. Sensing Performances toward Ammonia Exposure: Effect of the Exposure Time

**Figure 7:**
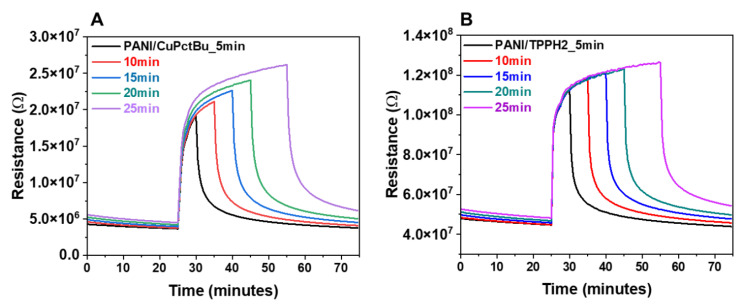
The resistive sensor response of PANI/CuPctBu (**A**) and PANI/TPPH_2_ (**B**) exposed to 300 ppm ammonia at different exposure times.

In this experiment, sensor layers have been exposed to a fixed ammonia concentration (300 ppm) while varying the exposure time. This experience will permit us to state whether the exposure time affects the response dynamics and diffusion into the layers. The secondary objective is to see whether or not the response time and recovery time are independent of the exposure time. The sensors were exposed to ammonia for 5, 10, 15, 20, and 30 min to perform this experiment, while keeping a fixed recovery time (set to 45 min) in dry air. The curves are presented in Figure 7. The figure shows the results for PANI/CuPctBu in Figure 7A, but the same behavior is observed for PANI/TPPH_2_ in Figure 7B.

One can see that the sensor displays a stable response variation between 10–30 min of exposure and relatively identical kinetics of adsorption–desorption. The sensors respond to ammonia quickly, and the steady state is rapidly obtained. This result shows that even if the sensing layers are pretty thick, diffusion into the layers is limited.

#### 3.2.3. Sensing Performances toward Ammonia Exposure: Effect of Humidity

Ammonia sensing with materials such as PANI is known to often experience the inconvenience of humidity as a limiting sensitivity factor. In fact, this is inherent to how ammonia sensing on the PANI layer is working. It is worth noting that PANI always contains some water molecules, even after drying [42]. The interaction mechanism with ammonia involves protonation and deprotonation, which can be affected by the presence of water. Consequently, humidity levels that are too high or too low can significantly affect the response.

**Figure 8:**
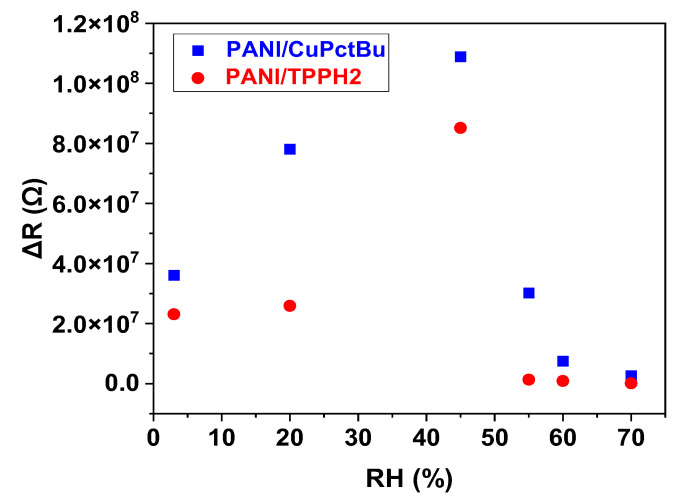
The sensor response of PANI/CuPctBu and PANI/TPPH_2_ exposed to 300 ppm ammonia at room temperature and different humidity levels.

Figure 8 displays the responses of the sensing devices exposed to 100 ppm of ammonia at different relative humidity levels. For RH between 15% and 70%, the responses of the PANI composites show almost the same tendency: the response continuously increases from 15% to 45% of RH and then decreases from 45% to 70%. For PANI/CuPctBu, the decrease in the response is relatively low, while PANI/TPPH2 shows a drastically reduced response when humidity increases. Such results can be explained by the behavior of humidity, which is a doping agent at a low water-concentration level and a structural reorganizing agent at a high water-concentration level.

The effect of humidity on ammonia gas sensing based on PANI blends or composites is a long-standing debate that has been discussed several times. This discussion is reasonable, since it is well known that PANI always contains water [43] (even after drying), and the conductivity of PANI increases in the presence of water vapor [44,45,46,47,48,49]. Apart from rare reported cases where PANI derivatives are not sensitive to humidity (via plasma treatment) [50], usually, the conductivity of PANI is affected by the presence of water vapor. It is also well accepted that polyaniline can be used as a sensitive film in a humidity sensor, and it allows measuring a wide range of humidity levels [46,48,49,51,52].

In many cases, the decrease of the resistance of polyaniline structures in either film, pellets, fibers, or blends when exposed to water vapor is overall observed [46,52,53]. In our case, the PANI is in a composite matrix where macrocycles surround the PANI and build a compact structure. It is expected that because of this compact structure, the layers of the sensor do not experience significant changes since the macrocycles used in this study are hydrophobic.

For PANI-based structures, the conductivity evolution with humidity is commonly attributed to the presence of water vapor, which acts as a proton donor and follows a proton transfer mechanism [45,48,53] and/or swelling [44,53,54]. Similar behavior has been observed for PANI-blend polymer films exposed to ammonia in humid environments where competitive sorption occurs [44]. For this study [44], it was reported that morphology also played a role. Such behavior has also been reported in cases where PANI nanostructures are exposed to only humidity [48].

However, when exposed to humidity and ammonia, the situation is different. In fact, instead of a monotonically decreasing response, we observed, as given in Figure 8, a bimodal response change, i.e., an increase of the response in the lower humidity range (up to 40% RH), and a decrease is observed within the higher humidity range (from 40% to 80% RH). Zeng et al. [45] even showed that this bimodal response change under humidity depends on the sensing layer morphology and found a correlation between PANI morphology (fibers or films) and the bimodal response confirmed by IR studies.

#### 3.2.4. Sensor Behavior at Lower Humidity Level: On/Off State

**Figure 9:**
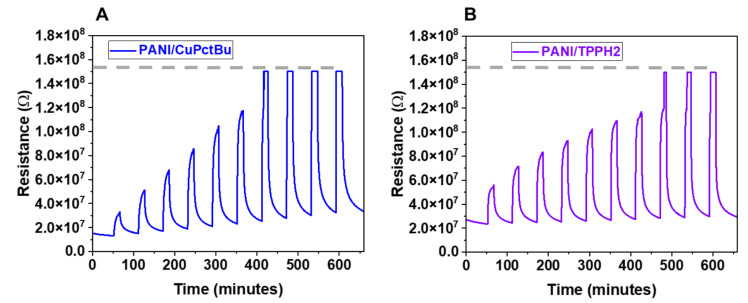
The room temperature sensor response of PANI/CuPctBu (**A**) and PANI/TPPH_2_ (**B**) exposed to 50–500 ppm ammonia (step of 50 ppm) at the lowest humidity level (<1% RH). Note: dashed lines represent the limit of the electrical device used to measure the resistance and is typically recorded for insulating materials.

We explained above that ammonia sensing with PANI materials often needs humidity. However, we wanted to probe the sensing behavior towards lower humidity (<1% relative humidity). In this experiment, very dried zero-grade air is used, and we keep the measured humidity between 0.3–0.6%. Figure 9 shows the results of the sensors under such low humidity conditions.

Both the PANI/MCs presented unusual behavior after beginning to respond to ammonia in the first exposure cycles, and then after some exposure/recovery cycles, they showed on/off states. The “ON” state represents the lower resistance value and the “OFF” the highest resistance value (here 1 × 10^38^ ohms). This off state is simply a high value of resistance that the electrical devices (Keithley) cannot measure. This off is, in fact, an isolating state. So, it seems that the PANI/MCs layers are conducting before the exposure, but once the ammonia enters the chamber, they become insulating. Overall, we can depict that the resistance under dry conditions is higher and the response is relatively higher. The situation is almost identical whatever the concentration. This result confirms the sensing mechanism as being humidity-dependent and is mainly attributed to the effect of the MCs on the PANI layers. After this experiment, we reran the same experiment to check the memory effect. The results are represented in Figure S5A (Supporting Information file) and illustrate the same tendency. We previously mentioned that wrapping the PANI with macrocycles that are insulating in nature has not had a limiting effect on the conductivity of the matrix. However, macrocycles potentially have an impact if measurements are performed at lower humidity levels. Considering that MCs are hydrophobic, they cannot ensure the role of the humidity reservoir to feed the PANI layer when operated in too-dry conditions. It is worth noting that the PANI layers experienced this lower humidity experiment without any on/off behavior (see Figure S5A in Supporting Information file); however, this response was higher compared to the PANI result at RH = 5%.

#### 3.2.5. Sensor Behavior at Different Operating Temperatures

**Figure 10:**
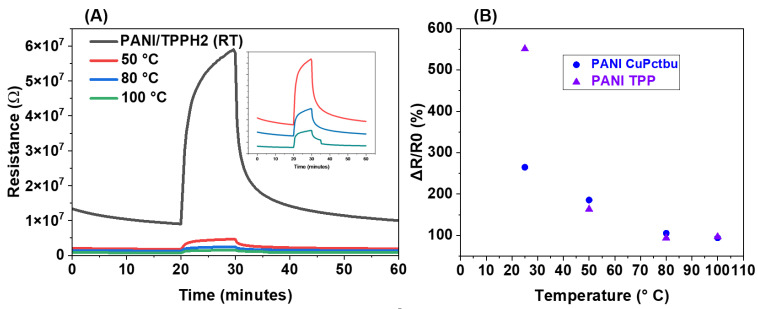
The sensor response of PANI/TPPH2 exposed to 300 ppm ammonia at different temperatures (**A**), and the corresponding response plotted as ΔR/R_0_ for PANI/CuPctBu and PANI/TPPH2 exposed to 300 ppm ammonia at different temperatures (**B**).

The effect of operating temperature on PANI blends or composites-based sensors dedicated to ammonia sensing can be a subject of concern since the temperature can also influence global resistance. Figure 10 represents the typical response–recovery characteristics recorded at different temperatures for PANI-based nanostructured composites exposed to 300 ppm ammonia. As stated previously, the study has been performed with sensors prepared from the PANI/MCs 2 dispersion preparation. Figure 10 shows that both sensors, PANI/CuPctBu and PANI/TPPH2, showed continuous decreasing responses over operating temperature.

We can explain the lower response by relying on the deprotonation/protonation mechanism. In fact, according to this mechanism, protonation leads to resistance decrease while deprotonation leads to resistance increase.

Yoshikawa et al. [55] have shown that temperature affects electronic properties by lowering the conductivity of the PANI composite. Kukla et al. [56], have conducted a similar study on electrochemically deposited PANI sensors and showed that response to ammonia monotonically decreases with temperature in the range of 25–80 °C.

#### 3.2.6. Cross-Sensitivity Experiment: Effect of the Interfering Gases

**Figure 11:**
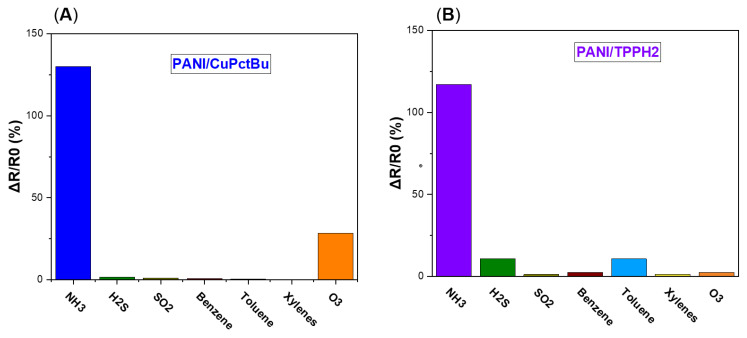
Cross sensitivity result for PANI/CuPctBu (**A**) and PANI/TPPH_2_ (**B**) exposed to ammonia (20 ppm), H_2_S (60 ppm), SO_2_ (60 ppm), benzene (>2000 ppm), toluene (>2000 ppm), xylene (1500 ppm), and O_3_ (1300 ppb).

Since the PANI is combined with aromatic macrocycles that can interact with VOCs such as benzene, toluene, and xylenes, the composites have been also exposed to VOCs (BTX) and other interfering gases. The relative responses of sensors towards NH_3_ as well as other interfering gases are reported in Figure 11. Although such macrocycles (CuPctBu and TPPH_2_) have shown potential as sensing materials for detecting VOCs, hybrid PANI/MCS are relatively insensitive to these analytes. The reason for such behavior can be attributed to the weak accessibility of the MCs in these composites for gas adsorption. Additionally, the low conductivity of these macrocycles and the lack of electronic charge transfer between VOCs and hybrid materials can be also limiting factors regarding the chemoresistive transduction of PANI/MCs nanostructures.

For the other interfering gases studied, sensitivity remains relatively weak even for high concentrations of the interfering gases compared to ammonia. The results demonstrate the good partial selectivity to ammonia achieved. Only ozone induces a non-negligible cross-sensitivity in PANI/CuPctBu. After several exposures to ozone, the sensors showed higher resistance variation that can be attributed to the irreversible degradation of the sensing layers. It is now well established that ozone involves strong chemisorption with phthalocyanine units, leading to the break of carbon-carbon double bonds, and so to the destructuration of gas adsorption sites.

#### 3.2.7. Sensing Performances toward Ammonia: Repeatability on Exposure Sequences

**Figure 12:**
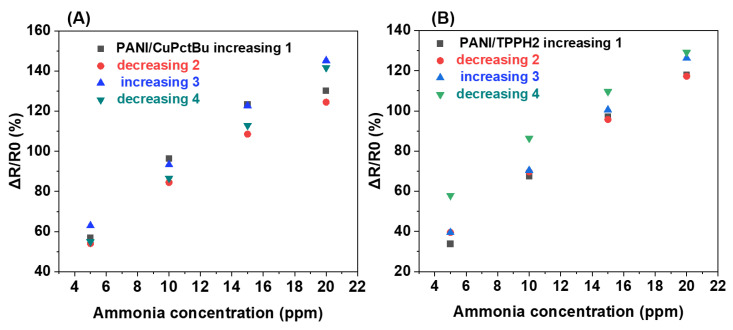
The sensor results obtained through repeated experimental sequences for PANI/CuPctBu (**A**) and PANI/TPPH_2_ (**B**).

In order to evaluate the repeatability level of the sensors, measurements were performed by exposing the layers to ammonia in a low concentration range (5–20 ppm). Two exposure sequences were applied: (a) an increasing concentration sequence from 5 to 20 ppm and, (b) a decreasing concentration sequence from 20 to 5 ppm. Thus, the effect of exposure history could, by this way, be taken into consideration in the response. The results depicted in Figure 12 reveal that sensors achieve repeatable responses to ammonia whatever the exposure sequence (increasing or decreasing concentrations).

The stability of the sensors and the repeatability of their responses showed that even though the presence of TPPH_2_ and CuPctBu on PANI results in a resistance increase, i.e., their inclusion gives rise to a more resistive structure, this goes without altering the sensing ability and the reactivity of the sensing layers. Even if the sensing layers are thicker than usual, a sign of mechanical instability has not been detected in the repeated exposure sequences. Again, this is a sign that these macrocycles wrapped on the PANI can serve as binding agents and ensure the achievement of a compact, highly durable structure.

## 4. Discussion

In the first experiment, we tried to find a relationship between the degree of functionalization and electrical characteristic. We have seen that the more the functional moieties (concentration), the lower the slope of the I–V curve, i.e., the higher the resistance of the devices. The intermediate preparation has been used to prepare sensors with the intent of avoiding devices with too high a resistance (approaching isolating state) or with too low a resistance (approaching that of PANI itself for low functional moieties). The response showed that this was a good compromise if lower humidity (<1% RH) is excluded from the test experiment. Characterization methods confirmed the effectiveness of the functionalization since both UV-Vis and Raman point out the existence of the composites. The responses recorded in the exposure time study show that even if the sensing layers are rather thick, gas diffusion into the layers can be limited.

In the humidity-dependent experiment, we observed bimodal behavior. In our case, the bimodal response can be explained as follows: in the first region (3% RH < humidity level < 50% RH), the response increase is mainly water adsorption on PANI since water acts as a proton source [48], therefore doping the conductivity. So, upon ammonia exposure in this region, the PANI sensing film doped by water is more sensitive to ammonia and, globally, a response increase is observed.

**Scheme 1:**
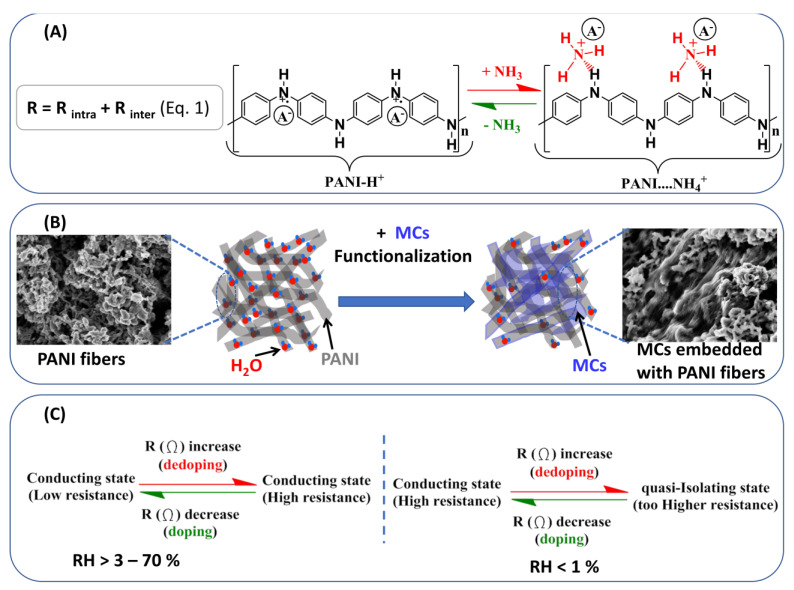
Illustrations showing the proposed sensing mechanism (**A**), highlighting the effect of water adsorption upon functionalization (**B**) and its consequence on the resistance evolution and the responses in different humidity environment (**C**).

Scheme 1 is given as support to explain and understand the general mechanism. Firstly, ammonia acts as deprotonating agent (de-doping) on conducting PANI and induces a resistance increase. Under air, the reverse reaction is produced, leading to protonation (doping). This explanation is summarized in Scheme 1A. Then, it is important to consider that adsorption of water molecules on PANI always occurs, even after drying [42]. Water adsorption in PANI induces two different and complementary behaviors. First, water acts as a proton source [48], meaning that moisture also affects the response. In the presence of minimal water content, the PANI itself is doped through protonation, and this doping improves the conductivity of the PANI. The second effect of a small moisture presence is to facilitate the inter-fibers junctions. In fact, water can be involved in inter-chain connections via hydrogen bonding [57]. The existence of hydrogen bonding, in some cases, leads to different nanostructures observed through self-assemblies [58,59]. If we consider the structure of PANI and PANI/MCs materials, we can easily understand that the resistance is a sum of the resistance of the fibers (intra) and the junction between fibers (inter) (see Equation (1) in Scheme 1A). However, it is evident that in the case of PANI/MCs (Scheme 1B), the intercalation of the MCs affects the resistance of the inter-fiber junctions and the global resistance.

In the cases of PANI/MCs, water molecules enhance the conductivity of the fibers (intra) through proton doping. Still, they can hardly facilitate the fiber-fiber (inter) junction due to the presence of the MCs (hydrophobic) that prevent the formation of connections or conducting paths. This case holds at relative humidity levels < 50%. In comparison, for higher relative humidity levels (>50%), the sensing film can absorb the ambient humidity, and the occurrence of the swelling phenomenon is accompanied by competitive sorption between water and ammonia.

As a consequence of this swelling, the inter-chain conductivity is affected and the global conductivity decreases, resulting in a decrease in the response when exposed to ammonia. However, one can easily imagine that the swelling phenomenon is limited in the presence of MCs in the PANI/MCs composites. Still, the competitive sorption limits the available adsorption sites and produces a decreasing tendency in the response. Araghi et al. [60] observed the same tendency upon studying the effect of humidity on the PANI/phthalocyanine composite used for the detection of CO_2_. They also pointed out the competitive sorption between water and CO_2_.

In the very low relative humidity regime (RH < 1%), the lack of water molecules can limit the doping action of water, giving rise to higher resistance. The action of ammonia to this structure is more drastic since it completely reacts with the few available sites through de-doping. This reaction gives rise to an extremely high film resistance (quasi-isolating state of the PANI/MCs (Scheme 1C)).

Wang et al. [47] showed that the resistance of PANI gradually increases with time, even if the relative humidity is kept constant. Moreover, this increase seems to be more important at higher RH levels. This result shows again that the swelling is a slow kinetic mechanism.

After exposure to higher humidity levels (the experiment performed in the 60–80% RH), the PANI/MCs sensitivity is lower than in the first exposure at 4–5% of RH. This result reveals that after swelling, desorption of moisture is a low kinetics mechanism. So, this result can be seen as a drawback when such sensors are exposed to higher humidity levels with time, affecting long-term sensitivity. This will be the subject of our next investigation.

In the experiment of ammonia exposure at different operating temperatures, we observed a continuous decrease in response from RT to 100 °C. We consider that heating the layer induces a loss of water molecules, and that humidity can act as a proton source. The temperature can also affect the polymer chain doping or reorganization [55]. So, heating the layers leads to a resistance decrease, as seen in the baseline resistance values (Figure 10). Zampetti et al. [61] conducted studies where the camphor-sulfonic acid-doped PANI composites showed a baseline current of increasing and then decreasing behavior when operating temperature was raised. However, they observed that the composites could be destroyed at high operating temperatures depending on the hosting polymer, leading to such a current decrease. In our case, the temperature experiments were performed in the RT to 100 °C range. In this range, the loss of dopant is essentially the significant contribution to conductivity [42]. This loss of dopant then explains the lower response observed when heating.

## 5. Conclusions

The combination of PANI with phthalocyanines and porphyrins has been achieved by an ultrasonic preparation method. The revealed composites present a compact structure with electrical behavior close to that of PANI, as given by the temperature-dependent electrical characteristics. The MCs seem to improve sensing performance while providing a partial selectivity towards other interfering gases. Their sensing performance is negatively affected at low humidity levels or after repeated high temperature exposure experiments. This is probably due to the presence of macrocycles, which are not good proton/water reservoirs. Overall, sensor performance is improved under normal humidity conditions (averaging 40–50% RH, in our lab) at room temperature. Highly resistive sensors can be fabricated with this method.

## Figures and Tables

**Table 1:** The sensing performances of PANI and PANI-based composites for ammonia, as reported in the literature.

Materials	Preparation	Range (ppm)	Response (at xx ppm)	Response Time	Recovery Time	Ref.
PANI/NiTSPc	Electrodeposition	5–2500	2.75 at 100 ppm	10 s	46 s	[35]
PANI/Poysiloxane	in situ polymerization	5–100	2.5 * (at 50 ppm)	208 s	263 s	[36]
Pyrolle/CoPc/CNTs	Drop casting	0.05–500	26.2% (at 50 ppm)	11.7 s	91.8 s	[37]
PA6/PANI	Paste	50–250	125% (at 200 ppm)	27 s	NA	[38]
PANI/ZnTPP	Electrodeposition	50–500	205.18% (at 500 ppm)	108 s	450 s	[39]
PANI/RGO	Spin coating	5–600	250% (at 100 ppm)	97 s	680 s	[40]
Nanostructured PANI	Spin coating	3–990	219% (at 990 ppm)	NA	NA	[41]
PANI/CuPctbu	Drop casting	5–500	529% (at 200 ppm)	510 s	990 s	This work
PANI/TPPH_2_	Drop casting	5–500	360% (at 200 ppm)	480 s	1080 s	This work

* Response = Rg/Ra (25% RH).

## Data Availability

Not applicable.

## Supplementary Materials

The following are available online at https://www.mdpi.com/article/10.3390/polym14050891/s1, Figure S1: Experimental test bench used for the ammonia sensing experiment, Figure S2: Current–voltage characteristics of the PANI/MCs composites at different MCs concentration and different voltage ranges, Figure S3: SEM image of PANI/TTPH2 showing some aggregates of TPPH2, Figure S4: Sensor response of: (A) PANI/CuPctBu (recorded after the first exposure cycles) and PANI (B) exposed to 50–500 ppm ammonia (step of 50 ppm) at the lowest humidity level (<1% RH).
