# Supplementary material for: Phthalocyanines and Porphyrins/Polyaniline Composites (PANI/CuPctBu and PANI/TPPH2) as Sensing Materials for Ammonia Detection
Source: Polymers (Basel). 2022 Feb 24;14(5):891. doi: 10.3390/polym14050891 (PMC8912817; doi:10.3390/polym14050891)
Supplement: Supplementary file 1 [file polymers-14-00891-s001.zip › polymers-1598474-supplementary.pdf]

polymers

MDPI

Supplementary Material

Phthalocyanines and Porphyrins/Polyaniline Composites (PANI/CuPctBu and PANI/$TPPH_2$) as Sensing Materials for Ammonia Detection

Alain Pauly [1], Sahal Saad Ali [1], Christelle Varenne [1], Jérôme Brunet [1], Eduard Llobet [2] and Amadou L. Ndiaye [3,*]

[1] Clermont Auvergne INP, CNRS, Institut Pascal, Université Clermont Auvergne, F-63000 Clermont-Ferrand, France; alain.pauly@uca.fr (A.P.); sahal@gmail.com (S.S.A.); christelle.varenne@uca.fr (C.V.); jerome.brunet@uca.fr (J.B.)
[2] Department of Electronic Engineering, Microsystems Nanotechnologies for Chemical Analysis (MINOS), Universitat Rovira i Virgili, 43007 Tarragona, Spain; eduard.llobet@urv.cat
[3] CNRS, Institut Pascal, Université Clermont Auvergne, F-63000 Clermont-Ferrand, France
* Correspondence: amadou.ndiaye@uca.fr; Tel.: +33-4734-07238

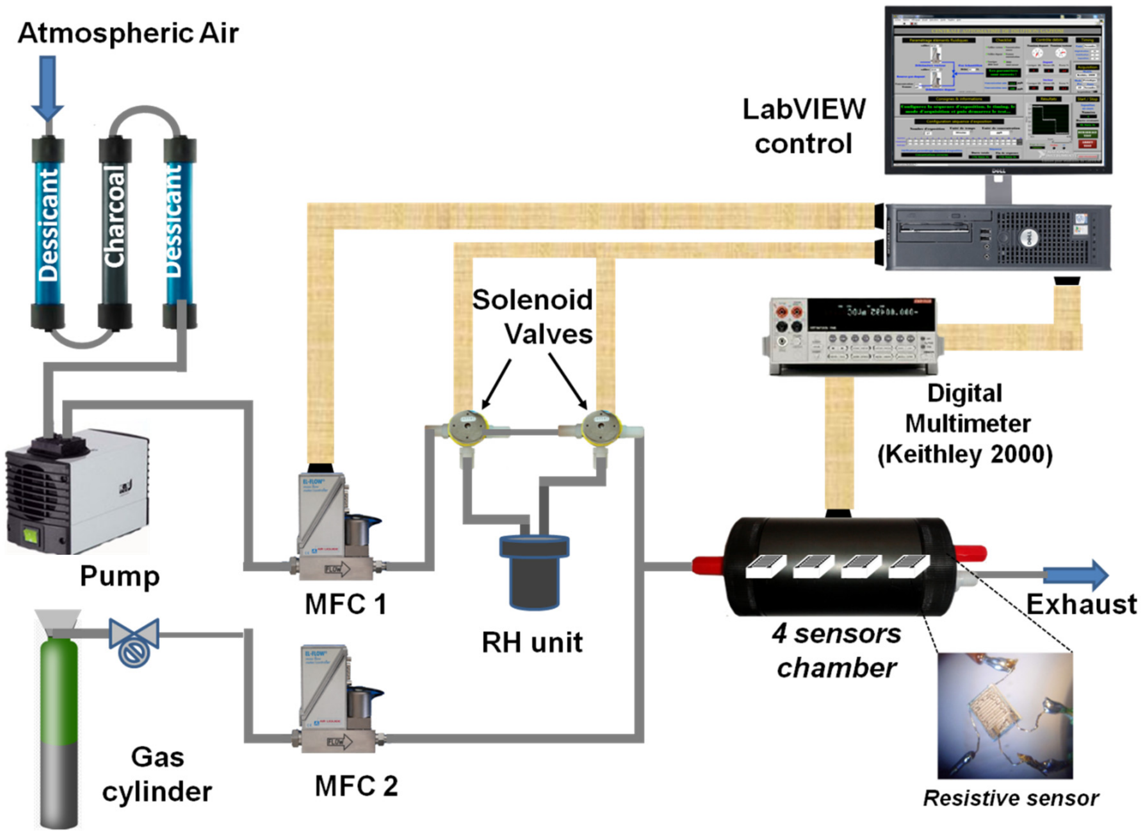

Figure S1. Experimental test bench used for the ammonia sensing experiment.

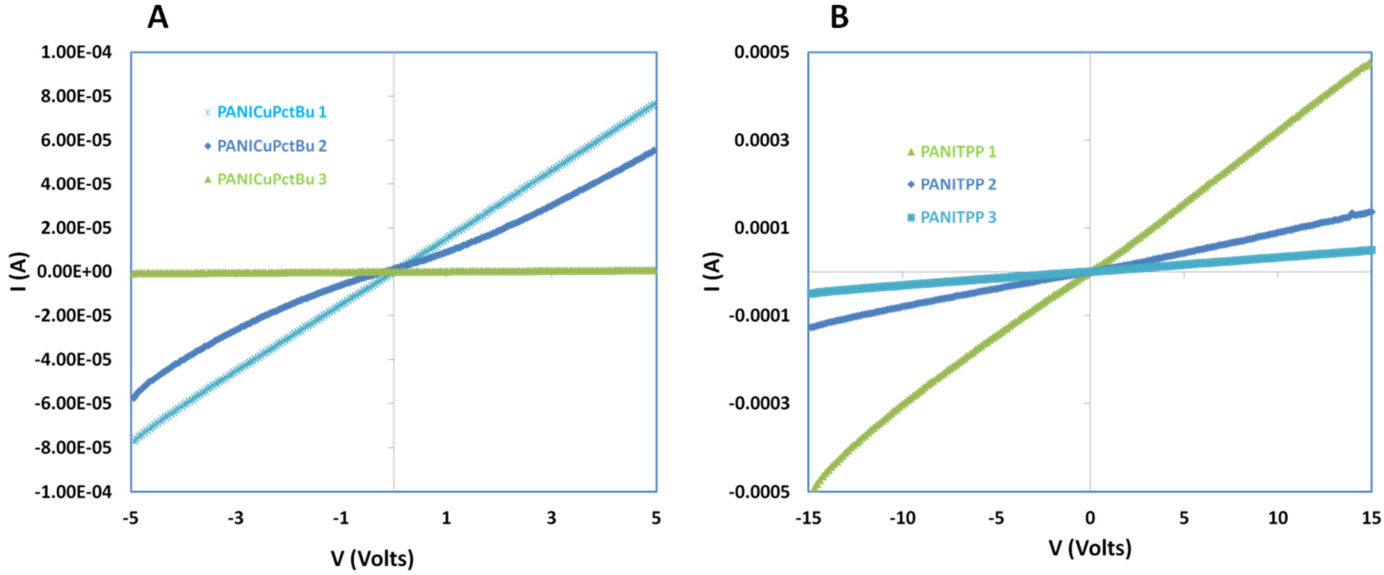

Figure S2. Current-voltage characteristics of the PANI/MCs composites at different MCs concentration and different voltage ranges.

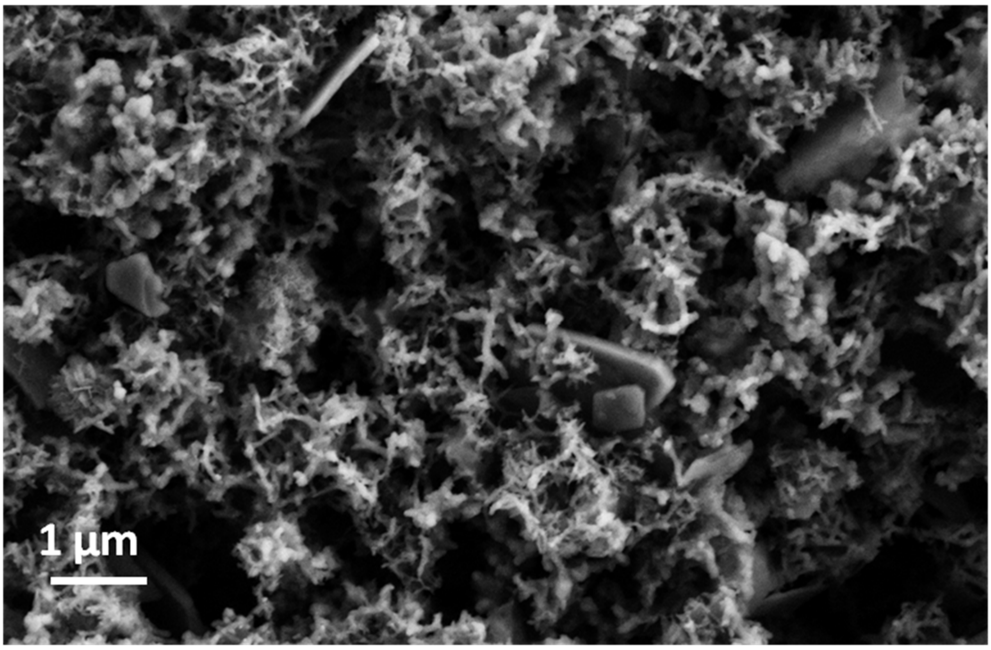

Figure S3. SEM image of the PANI/TTPH2 showing some aggregates of $TPPH_2$.

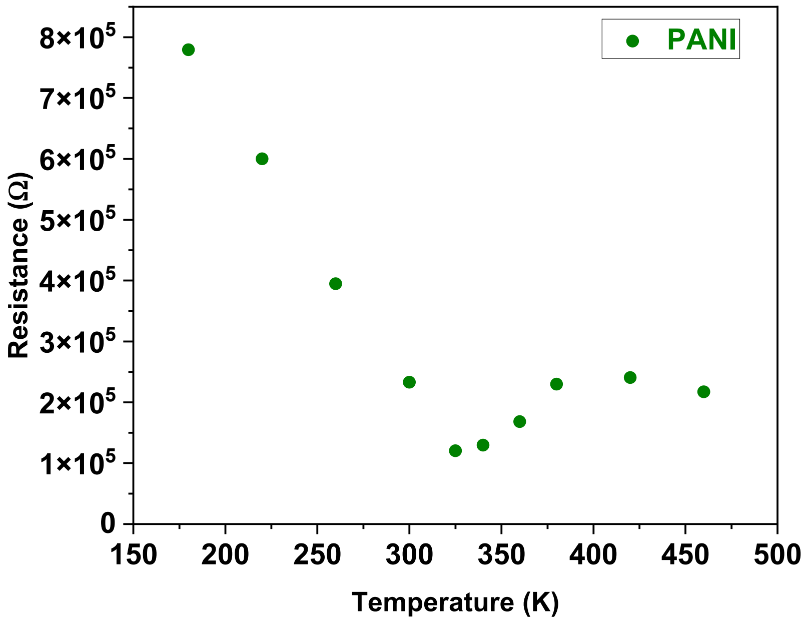

1

Figure S4. Temperature-dependent resistance variation for PANI. Resistance are calculated from current-voltage measurement at the given temperature.

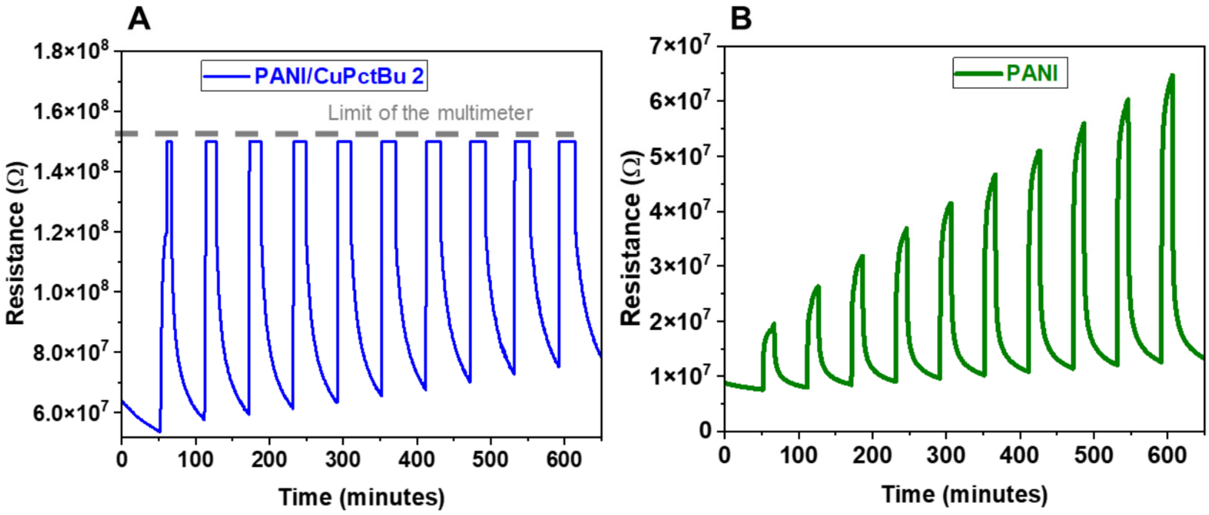

Figure S5. Sensor response of: (**A**) PANI/CuPctBu (recorded after the first exposure cycles) and PANI (**B**) exposed to 50–500 ppm ammonia (step of 50 ppm) at lowest humidity level (<1% RH).
